# Neurological Disorders in Dogs: A Retrospective Analysis of Prevalence, Aetiology, Lesion Locations, and Regional Variations in Northeastern Iran

**DOI:** 10.1002/vms3.70952

**Published:** 2026-04-28

**Authors:** Ali Behrouzian, Ali Asghar Sarchahi, Hadi Jabbari Noghabi

**Affiliations:** ^1^ Faculty of Veterinary Medicine Ferdowsi University of Mashhad Mashhad Iran; ^2^ Department of Clinical Sciences, Faculty of Veterinary Medicine Ferdowsi University of Mashhad Mashhad Iran; ^3^ Department of Statistics, Mathematics Ferdowsi University of Mashhad Mashhad Iran

**Keywords:** dog, nervous system, neurological disorders, retrospective study, seizure, spinal trauma

## Abstract

**Background:**

Neurological disorders in dogs are a significant concern in veterinary medicine and affect various breeds and ages. Previous studies have reported differing prevalence rates and aetiologies across regions, highlighting the need for a comprehensive epidemiological understanding to enhance diagnostic and treatment strategies.

**Objectives:**

This retrospective study aimed to investigate the prevalence and frequency of neurological disorders in dogs referred to the Veterinary Teaching Hospital.

**Methods:**

This study analyzed records from 13,430 patients referred to the hospital. Among these, 9131 cases involved dogs. Of these, 422 dogs had neurological disorders, and their records were analyzed for the study.

**Results:**

The prosencephalon was the most commonly affected location (81 cases, 19.6%), followed by multifocal involvement (62 cases, 15%) and the T3‐L3 spinal cord (56 cases, 13.5%). Infectious diseases, especially canine distemper diseases, were the most common cause, accounting for 136 cases (33.2%). Unknown causes ranked second (66 cases, 15.9%), with trauma third (56 cases, 13.3%). Other neurological disorders included intervertebral disc displacement, polyradiculoneuritis, vestibular injury, and poisoning. Notably, although most affected dogs (81.3%) were under 5 years of age, the relative prevalence of neurological disorders increased with advancing age.

**Conclusion:**

Infectious diseases, especially canine distemper, trauma, and unknown causes, are the primary contributors to neurological disorders in this region. The observed high prevalence of these conditions contrasts with the findings from other regions, emphasizing the need for improved prevention and treatment strategies. Furthermore, many neurological disorders affecting the prosencephalon can be effectively diagnosed and managed through basic neurological examinations without necessitating advanced imaging techniques.

## Graphical abstract

This retrospective study evaluated 422 dogs with neurological disorders among 9131 canine referrals over 8.5 years in northeastern Iran. Infectious diseases, particularly canine distemper (33.2%), were the leading cause, followed by trauma and idiopathic conditions. The prosencephalon was the most frequently affected anatomical region. Most cases were diagnosed using basic clinical and neurological examinations. These findings highlight the importance of preventive vaccination and structured neurological assessment in resource‐limited veterinary settings.

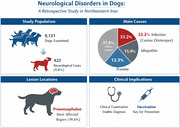

## 1 Introduction

Neurological disorders represent a significant proportion of clinical cases in small animal veterinary practice and remain among the most diagnostically challenging conditions encountered in dogs. These disorders can affect animals of various breeds and ages and encompass a wide spectrum of aetiologies, including infectious, inflammatory, degenerative, traumatic, metabolic, congenital, and idiopathic conditions (Elbert et al. 2022; Ettinger et al. 2024; Fluehmann et al. 2006; Gómez‐Flores et al. 2021; Kim et al. 2019; Mayousse et al. 2017). Clinical manifestations range from seizures and altered mentation to gait abnormalities, paresis, paralysis, and behavioural changes, often resulting in substantial morbidity or mortality if not promptly diagnosed and managed. While extensive epidemiological studies from Europe, North America, and East Asia have described the prevalence and distribution of canine neurological diseases (Elbert et al. 2022; Fluehmann et al. 2006; Gómez‐Flores et al. 2021; Kim et al. 2019; Mayousse et al. 2017; Tipold 1995), large‐scale epidemiological data from the Middle East and neighbouring regions are relatively limited (Mousafarkhani et al. 2023). This scarcity of regional data poses a significant challenge for clinicians, as extrapolation from Western or Asian populations may not accurately reflect local disease patterns due to differences in vaccination coverage, access to advanced diagnostics, environmental exposures, and dog population structure. Recent studies from the Middle East provide valuable insight into region‐specific patterns of neurological disease in dogs. In Turkey, retrospective analyses have consistently shown that canine distemper virus (CDV) remains a major cause of neurological disease, particularly in younger and unvaccinated populations, presenting clinically with ataxia, seizures, and demyelinating lesions of the central nervous system (Gülersoy et al. 2023). Similarly, studies from Iran have documented endemic CDV infection as a significant contributor to neurological morbidity among clinical cases, underscoring the impact of preventable infectious aetiologies (Mousafarkhani et al. 2023). Importantly, a study conducted in Egypt found that among dogs presenting with meningeo‐encephalitis, canine distemper virus was the most frequently recorded cause, while other identified factors included parasitic agents and head trauma as an important non‐infectious contributor to neurological disease (Abdel‐Saeed and Elmashad 2020). This pattern of etiological distribution shows notable similarity to the findings of the present study, where CDV emerged as a leading cause and traumatic head injury ranked among the common aetiologies.

Collectively, these regional reports highlight a pattern in which infectious agents—particularly CDV—play a dominant role in canine neurological disease in the Middle Eastern context, while traumatic and parasitic causes also contribute significantly. Moreover, some neurological conditions may indirectly affect human health, especially those linked to zoonotic pathogens (Bear et al. 2020). However, comprehensive, long‐term epidemiological studies that characterize the relative prevalence, demographic risk factors, lesion localization, diagnostic approaches, treatment outcomes, and prognostic data in this region remain relatively scarce. This gap underscores the need for locally derived data to inform evidence‐based clinical decision‐making and the development of region‐specific diagnostic and therapeutic strategies, particularly in settings with limited access to advanced imaging modalities such as magnetic resonance imaging (MRI) and computed tomography (CT).

Given that neurological disorders account for a significant proportion of clinical cases in small animal practice (Fluehmann et al. 2006; Gómez‐Flores et al. 2021; Mayousse et al. 2017), there is a clear need to better understand their epidemiology, presentation, and treatment response. Therefore, this retrospective study aimed to investigate the prevalence, etiological spectrum, anatomical lesion localization, demographic characteristics (e.g., age, sex, breed, housing, and diet), diagnostic approaches, therapeutic strategies, and clinical outcomes of dogs with neurological disorders referred to a major veterinary hospital in northeastern Iran over an 8.5‐year period. By contextualizing study findings within the broader Middle Eastern literature, this research seeks to provide novel, regionally relevant epidemiological insights that will support more effective clinical management and improve outcomes for dogs suffering from neurological disorders in similar settings.

## 2 Materials and Methods

### 2.1 Study Design and Data Collection

This retrospective study was approved by the Research Council of Ferdowsi University of Mashhad (grant No. 61120). As the research involved only the analysis of existing, fully anonymized patient records, and no new interventions or direct animal contact, it was exempt from institutional animal care and use committee approval. All data were extracted from the Veterinary Teaching Hospital's internal medical record system and were accessible only to authorized researchers. Identifiable information was removed prior to analysis to ensure confidentiality. Written informed consent for the use of anonymized clinical data for research purposes was obtained from all animal owners at the time of hospital admission through the standard hospital consent procedure. Medical records of dogs diagnosed with neurological disorders and referred to the Veterinary Teaching Hospital of Ferdowsi University of Mashhad between 21 March 2015 and 21September 2023 were reviewed. Data were collected from both paper files and electronic records within the small animal internal medicine department. The extracted information included type of neurological disease, anatomical location of the lesion, treatment response, and demographic factors such as age, sex, breed, weight, vaccination status, housing conditions (indoor/outdoor), lifestyle (working, companion, stray), relevant medical history, and diet.

### 2.2 Diagnostic Methods

A variety of diagnostic methods have been employed to identify the underlying cause of neurological disorders. These included neurological and physical examinations, complete blood counts (CBCs), serum biochemical tests, cerebrospinal fluid (CSF) analysis, radiography, ultrasound imaging, myelography, CT scans, and MRI. Each method plays a crucial role in localizing neurological lesions and determining the underlying causes of diseases. Dogs suspected of having neurological canine distemper were identified based on at least two classical signs such as myoclonus, seizures, nasal or ocular discharge, gastrointestinal symptoms, or hyperkeratosis. Confirmation of distemper virus infection was made through either rapid immunochromatographic diagnostic kits applied to conjunctival or CSF samples or through reverse‐transcription polymerase chain reaction testing of whole blood or CSF. Due to limitations in resources and cost, PCR testing was not performed in all suspected cases. In such instances, a positive rapid test combined with compatible clinical signs was considered adequate for diagnosis. Cases with clinical suspicion but negative or unavailable laboratory tests were not included as confirmed distemper.

### 2.3 Treatment Methods

The treatment approaches utilized in this study included medical therapy, surgical procedures, and adjunctive therapies. The response to these treatments was evaluated based on follow‐up data, clinical improvement, and duration of medication. Additionally, the analysis considered how different factors, such as age, sex, and breed, influenced treatment outcomes. While treatment regimens for specific neurological diseases (such as distemper) were generally based on standard protocols, including supportive care, antibiotics, and vitamin supplementation, there was some variation in the exact medications and dosages used, reflecting individual patient needs and clinical judgment. This variability is a recognized limitation of retrospective studies and is discussed accordingly.

To assess breed‐specific susceptibility to neurological disorders, we calculated the proportion of affected cases relative to the total number of dogs of each breed presented to the hospital during the study period. Breeds with fewer than 50 referrals were excluded from this analysis to reduce bias due to small sample sizes.

### 2.4 Statistical Analysis

All statistical analyses were performed using SPSS version 24 (IBM Corp., Armonk, NY, USA) and Microsoft Excel. Because this study retrospectively evaluated all canine neurological cases referred to the hospital during the defined study period (rather than a randomly selected sample), the primary analytical approach was descriptive epidemiology. Frequencies and percentages were calculated to summarize categorical variables such as breed, sex, age group, lesion localization, and etiological category, diagnostic modality, treatment type, and clinical outcome. Disease prevalence was calculated as the proportion of neurological cases relative to the total number of referred dogs within the same category (e.g., age group or breed) during the study period. Inferential statistical analyses were performed to explore potential associations between selected categorical variables. Chi‐square tests were used to evaluate relationships between categorical factors (e.g., breed and neurological disease prevalence, age group, and etiological category). Fisher's exact test was applied when expected cell counts were less than five. Correlation analysis (Phi coefficient) was used to assess associations between binary variables where appropriate.

A significance threshold of *p* < 0.05 was adopted for inferential analyses. No adjustments for multiple comparisons (e.g., Bonferroni correction) were applied. This decision was made because the study population comprised the entire cohort of referred cases during the study period, and the inferential analyses were exploratory rather than hypothesis‐driven. Therefore, statistical findings should be interpreted cautiously, with emphasis placed primarily on descriptive patterns rather than isolated *p* values. For variables with missing data, percentages were calculated using available‐case denominators. The number of recorded observations is reported for each variable to enhance transparency.

## 3 Results

### 3.1 General Characteristics of the Studied Dog Population

This retrospective study included 13,430 animals referred to the Veterinary Teaching Hospital of Ferdowsi University of Mashhad from 21 March 2015 to 21 September 2023. Among these, 9131 (68%) were dogs. Among the total dog referrals, 4531 (49.6%) were for routine checkups and vaccinations, whereas 4600 (50.4%) were for various medical conditions. Notably, 422 dogs (4.6% of total dog referrals and 9.2% of sick dogs) were diagnosed with neurological diseases. These findings reflect the burden of neurological disease within the referred canine population during the 8.5‐year study period.

The general characteristics of the dog population in this study, including age, sex, sterilization status, breed, season, vaccination status, and diet, are presented in Table 1. The average age of the dogs diagnosed with neurological diseases was 24 ± 33 months. The dogs were categorized into four age groups: under 6 months, 6–12 months, 12–60 months, and over 60 months. Age was recorded for 400 of the 422 dogs (94.8%), whereas the age of 22 dogs (5.2%) was not documented. Among the 400 dogs with recorded age data, the majority (166/400; 41.5%) were between 12 and 60 months old. However, when calculating prevalence relative to total referrals within each age group, dogs over 60 months of age showed the highest prevalence of neurological disorders (8.8%). The association between age group and neurological disease prevalence showed a moderate effect size (phi = 0.239). In terms of sex distribution among the 422 dogs diagnosed with neurological disorders, sex was recorded for 419 cases (99.3%), while three dogs (0.7%) lacked documentation. Among dogs with recorded sex, 243 (58%) were male and 176 (42%) were female. No statistically significant associations were found between sex and the prevalence of neurological diseases (*p* > 0.05), with a prevalence of 4.8% in males and 4.6% in females. Marked differences in the distribution of neurological disorders were observed across sex, age groups, and breeds. Infectious diseases were more common in males (84 cases, 61.8%) and predominantly affected younger dogs under 12 months of age (84 cases, 63.2%). Traumatic causes also showed a higher prevalence in males (34 cases, 60.7%) and affected both young and adult dogs. Intervertebral disc disease was more frequent in females (17 cases, 54.8%) and mainly diagnosed in older dogs over 12 months (27 cases, 87.1%). Fibrocartilaginous embolism occurred more often in females (11 cases, 61.1%) and in adult dogs aged 12–60 months (11 cases, 64.7%). Breed‐wise, Kuchi dogs represented the majority of infectious (51 cases) and traumatic (12 cases) cases, while Terrier (7 cases), Spitz (3 cases), and Shih Tzu (4 cases) breeds were more commonly affected by idiopathic epilepsy and intervertebral disc disease. Some causes, such as acute polyradiculoneuritis (26 cases), showed no significant differences in distribution across sex, age, or breed. These findings highlight the influence of sex, age, and breed on the prevalence of specific neurological disorders in this population. Vaccination status was documented for 279 of the 422 dogs (66.1%), whereas records were unavailable for 143 dogs (33.9%). Among dogs with available vaccination data (n = 279), 172 (61.6%) were fully vaccinated, 24 (8.6%) had incomplete vaccination, and 83 (29.7%) were unvaccinated. Additionally, dietary information was recorded for 346 dogs (82.0%), while it was missing for 76 (18.0%). Among dogs with available records (n = 346), 266 (76.9%) were fed homemade diets, 64 (18.5%) received mixed diets, and 16 (4.6%) were fed exclusively commercial diets. There were no significant differences in the incidence of neurological diseases across different seasons (phi = 0.018). However, the highest prevalence was observed in spring (5%), whereas the lowest occurred in summer (4.1%).

**TABLE 1:** General characteristics of the 422 dogs evaluated in the present study.

Variable	Frequency	Relative frequency (%)	Total referred dogs	Prevalence of neurological disease (%)
**Age**				
Under 6 months	95	23.8	3855	2.5
Between 6 and 12 months	62	15.5	1034	6.0
Between 12 and 60 months	166	41.5	2585	6.4
Over 60 months	77	19.2	876	8.8
Total	400	100.0	8750	
Not recorded	22	5.2	381	5.8
**Sex**				
Male	243	58.0	5094	4.8
Female	176	42.0	3865	4.6
Total (recorded)	419	100.0	8959	
Not recorded	3	0.7	357	0.8
**Sterilization status**				
Sterilized	64	20.6	—	—
Not sterilized	246	79.4	—	—
Total (recorded)	310	100.0	—	—
Not recorded	112	26.5	—	—
**Vaccination status**				
Fully vaccinated	172	61.6	—	—
Incomplete vaccination	24	8.6	—	—
No vaccination	83	29.7	—	—
Total (registered)	279	100	—	—
Not registered	143	33.9	—	—
**Diet**				
Home diet	266	76.9	—	—
Mixed home and commercial	64	18.5		—
Commercial	16	4.6	—	—
Total (registered)	346	100.0	—	—
Not registered	76	18.0	—	—
**Season**				
Spring	111	26.3	2199	5.0
Summer	88	20.9	2144	4.1
Autumn	114	27.0	2310	4.9
Winter	109	25.8	2478	4.4
Total	422	100.0	9110	—

### 3.2 Prevalence of Neurological Diseases by Breed

Table 2 presents the prevalence of neurological diseases by breed. To minimize bias associated with small sample sizes, breeds with fewer than 50 referrals (e.g., Papillon, French Bulldog, Maltese, Golden Retriever, Cocker Spaniel, Pit Bull, Pug, Dalmatian, and Miniature Pinscher) were excluded from the breed‐based analysis. A significant association was found between breed and the prevalence of neurological diseases (phi = 0.141). Compared with other breeds, the Kochi, Pekingese, Great Dane, and German Shepherd breeds presented a greater prevalence of neurological diseases. In contrast, the Poodle and Shih Tzu breeds presented lower prevalence rates.

**TABLE 2:** Prevalence of neurological diseases by breed during the study period.

Breed	Frequency	Relative frequency (%)	Total frequency of referred dogs	Disease prevalence (%)
Kochi	101	24.8	876	11.5
Pekingese	11	2.7	155	7.1
Great Dane	3	0.7	56	5.4
German Shepherd	29	7.1	553	5.2
Husky	16	3.9	328	4.9
Rottweiler	3	0.7	68	4.4
Terrier	49	12.0	1181	4.1
Doberman	8	2.0	203	3.9
Mix	82	20.1	2307	3.6
Spitz	40	9.8	1116	3.6
Pomeranian	11	2.7	343	3.2
Maltese	1	0.2	34	2.9
Poodle	2	0.5	76	2.6
Shih Tzu	21	5.1	836	2.5
Sarabi	2	0.5	103	1.9
Papillon	2	0.5	3	66.7
Miniature Pinscher	1	0.2	3	33.3
French Bulldog	5	1.2	22	22.7
Golden Retriever	4	1.0	41	9.8
Cocker Spaniel	3	0.7	35	8.6
Other	14	3.4	380	3.7
Total	337	100	8753	—

### 3.3 The Exact Location of Neurological Disease

Table 3 shows the distribution of neurological disease locations in the referred dogs during the study period. The anatomical location of neurological lesions was recorded for 414 of the 422 dogs (98.1%), while the lesion site was not documented in 8 cases (1.9%). The prosencephalon was the most common area affected by neurological disease, with 81 out of 414 dogs (19.6%) showing involvement. Notably, 27 of these cases (33.3%) were associated with epilepsy. The mixed‐breed dogs presented the highest prevalence of prosencephalon involvement, accounting for 18 cases (22.2%). In addition to the prosencephalon, multifocal involvement was identified in 62 patients (15%), with distemper diagnosed in 40 of these patients. Lesions in the spinal cord (total cases: 188) were distributed as follows: T3‒L3 spinal cord (56 cases, 13.5%), L4‒S3 spinal cord (40 cases, 9.7%), total spinal cord (45 cases, 10.7%), C6‒T2 spinal cord (27 cases, 6.5%), and C1‒C5 spinal cord (20 cases, 4.8%). Additionally, the peripheral nervous system accounted for 32 patients (7.7%), whereas brainstem involvement was observed in 11 patients (2.7%).

**TABLE 3:** Distribution of neurological disease locations in dogs.

Location of involvement	Frequency	Relative frequency (%)
Prosencephalon	81	19.6
Multifocal (more than one area involved)	62	15.0
T3‐L3 spinal cord	56	13.5
Total spinal cord	45	10.7
L4‐S3 spinal cord	40	9.7
Peripheral nervous system	32	7.7
C6‐T2 spinal cord	27	6.5
Vestibular system	27	6.5
C1‐C5 spinal cord	20	4.8
Brainstem	11	2.7
Cerebellum	8	1.9
Midbrain	5	1.2
Total	414	100
Not recorded	8	1.9

### 3.4 Causes of Neurological Diseases During the Study Period

The underlying causes of neurological diseases in the 422 dogs included in the present study are listed in Table 4. Infectious diseases were the predominant cause, accounting for 136 cases (33.2%). The second most common cause was classified as unknown, with 66 cases (15.9%). Trauma emerged as the third most significant cause, accounting for 56 cases (13.3%). Other major causes included intervertebral disc disease (32 cases, 7.6%) and fibrocartilaginous embolism (18 cases, 4.3%). The infectious diseases contributing to neurological conditions in this study included distemper, parvovirus, ear infections, septicaemia, meningitis, and gastrointestinal involvement. Notably, distemper was the most frequently diagnosed infectious disease, affecting 112 dogs. Importantly, ‘suspected rabies’ in this study refers to clinical signs consistent with rabies; however, a definitive diagnosis was not confirmed.

**TABLE 4:** Causes of neurological diseases in dogs during the study period.

Cause of disease	Frequency	Relative frequency (%)
Infection	136	33.2
Unknown	67	15.9
Trauma	56	13.3
Disc disease	32	7.6
Idiopathic epilepsy	27	6.4
Acute polyradiculoneuritis	26	6.2
Poisoning (organophosphates [11], rodenticides [2], lead [2 cases], and unspecified [3])	18	4.3
Fibrocartilaginous embolism	18	4.3
Congenital	10	2.4
Tumour (brainstem [3], T3‐L3 spinal cord [2] and unknown [1])	6	1.4
Cauda equina syndrome	5	1.2
Behavioural problems	5	1.2
Botulism	4	0.9
Idiopathic tremor	4	0.9
Diabetes mellitus	2	0.5
Anaemia	1	0.2
Vaccine	1	0.2
Foreign body	1	0.2
Myasthenia gravis	1	0.2
Hepatoencephalopathy	1	0.2
steroid responsive meningitis arteritis	1	0.2
Total recorded	422	100

### 3.5 Diagnostic Methods Used to Diagnose Various Neurological Diseases

Table 5 summarizes the diagnostic methods employed to identify neurological diseases in referred dogs during the study period. Diagnostic methods were documented for 366 dogs (86.7%), whereas this information was missing for 56 dogs (13.3%). Percentages shown in Table 5 are calculated based on the 366 cases with available diagnostic records. In 27.9% of the patients, neurological disease was diagnosed solely through a combination of general and neurological examinations. In 21.1% of the instances, a diagnosis was achieved via neurological examination and a CBC, whereas 21.9% of the patients were diagnosed via both neurological and radiological examinations. Importantly, 87.3% of the neurological cases were diagnosed via readily accessible methods, including general and neurological examinations, CBC, radiography, and myelography. Radiography was utilized in 44% of the patients, CBC in 43.8%, and biochemistry panels in 6.6%. MRI has been used less frequently due to its limited availability and relatively high cost. Necropsy was infrequently performed, primarily due to owners’ refusal or instances where the dog did not pass away within the clinic.

**TABLE 5:** Diagnostic methods used to identify neurological diseases during the study period.

Diagnostic methods	Frequency	Relative frequency (%)
Neurological examination	72	19.7
Ear examination	1	0.3
Neurological examination + radiography	80	21.9
Neurological examination + CBC	81	22.1
Neurological examination + CBC + radiography	24	6.6
Neurological examination + MRI + radiography	1	0.3
Neurological examination + radiography + myelography	10	2.7
Neurological examination + ear examination + biochemical analysis	5	1.4
Neurological examination + general examination (excluding neurology and ear) + radiography + CBC	22	6.0
General examination	2	0.5
Neurological examination + general examination (excluding neurology and ear)	28	7.7
Neurological examination + necropsy	1	0.3
Neurological examination + necropsy + CBC + biochemistry + radiography	2	0.5
General examination + CBC + biochemical analysis	5	1.4
Neurological examination + general examination + radiography	2	0.5
Neurological examination + general examination + radiography + CBC + biochemical analysis	8	2.2
Neurological examination + general examination + CBC	6	1.6
Neurological examination + CBC + biochemical analysis + ultrasonography	4	1.1
Comprehensive diagnostic approach	12	3.3
Total registered	366	100
Not registered	56	13.3

### 3.6 Treatments Used

Table 6 presents the various treatments administered to dogs with neurological diseases in this study. Treatment information was recorded for 371 dogs (87.9%), while treatment data were unavailable for 51 dogs (12.1%). Percentages in Table 6 are calculated using the 371 cases with documented treatment information. Corticosteroids (including prednisolone, dexamethasone, and betamethasone) were the most commonly prescribed treatments, given to 183 patients (49.2%), of whom 101 (27.2%) received them alone. Among patients treated solely with corticosteroids, a 64.7% improvement rate was observed (22 out of 34 patients with recorded follow‐up), whereas the rate of no improvement was 35.3% (12 out of 34 patients). These findings suggest that corticosteroids can be effective treatment options for dogs with certain neurological diseases. In this study, a total of 74 patients (20%) received anticonvulsant medications. Among these patients, 38 patients (51.4%) also received corticosteroids, and 23 patients (31.1%) received antibiotics. The recovery rate for patients treated exclusively with anticonvulsants was 69% (11 out of 16), indicating their potential effectiveness for selecting neurological conditions. Importantly, these results are based on the outcomes of improvement or lack thereof in 158 patients with recorded follow‐up data during the study period.

**TABLE 6:** Types of treatments used in dogs during the study period.

Treatment types	Frequency	Relative frequency (%)
Corticosteroid	101	27.2
Osmotic	1	0.3
Analgesic	2	0.5
Antibiotic	10	2.7
Corticosteroid + analgesic + antibiotic	5	1.4
Corticosteroid + antibiotic	22	5.9
Osmotic + antibiotic	9	2.4
Diuretic + osmotic	3	0.8
Anticonvulsant	34	9.2
Diuretic + osmotic + corticosteroid	4	1.1
Anticonvulsant + corticosteroid + osmotic	12	3.2
Rest	10	2.7
Corticosteroid + osmotic + antibiotic	9	2.4
Corticosteroid + diuretic	4	1.1
Euthanasia	29	7.8
Refer to surgery	21	5.6
Anticonvulsant + osmotic + antibiotic + anticoagulant	2	0.5
Corticosteroid + anticonvulsant + diuretic	5	1.3
Anticonvulsant + corticosteroid + antibiotic	13	3.5
Anticonvulsant + osmotic + corticosteroid + antibiotic + diuretic	8	2.1
Withdrawal from treatment	9	2.4
Other Treatments	58	15.6
Total registered	371	100
Not registered	51	12.1

### 3.7 Prognosis of Neurological Patients in Referred Dogs

Prognostic assessment was documented for 306 of the 422 dogs (72.5%), while information was missing for 116 dogs (27.5%). The prognosis of neurological patients varies significantly. Among the assessed cases (*n* = 306), 75 dogs (24.5%) had a good prognosis, whereas 142 dogs (46.4%) had a poor prognosis. A cautious prognosis was assigned to 88 patients (28.8%).

### 3.8 Recovery Rates of Neurological Patients Among the Dogs Included in the Present Study

Follow‐up outcome data were available for 158 of the 422 dogs (37.4%), whereas no follow‐up information was recorded for 264 dogs (62.6%). Among the 158 patients with available follow‐up data, 71 patients (45.6%) achieved complete recovery, while 86 patients (54.4%) showed no improvement at the time of assessment. Factors contributing to a lack of recovery may include inadequate owner compliance with treatment recommendations, such as inconsistent medication administration or incomplete treatment courses, as well as failure to return for recommended follow‐up examinations.

## 4 Discussion

This retrospective study analyzed 422 dogs with neurological disorders referred to the Veterinary Teaching Hospital of Ferdowsi University of Mashhad over 8.5 years, revealing an average of approximately 50 cases per year. In a similar study conducted by Mayousse et al. (2017) over a 14‐year period (2002–2016), 3846 French bulldogs were referred to veterinary hospitals in France, with 343 (8.9%) diagnosed with neurological diseases. The overall prevalence of neurological diseases in this population was 9.2%, comparable to the 8.9% reported by Mayousse et al. (2017) in a study of French bulldogs. While Mayousse et al. focused on a single breed, this study examined a more diverse population. Notably, French bulldogs in this study had a higher prevalence of neurological disorders (22.7%) than those reported by Mayousse et al. However, the smaller sample size of French bulldogs in this study may influence these findings. In a retrospective study conducted in Guatemala by Gomez Flores et al. (2021), 95 dogs with neurological diseases were identified and examined over 1 year (2017). The present study identified and examined approximately 50 neurological patients annually, indicating that Gomez Flores et al. reported a relatively large number of cases per year. In another retrospective study, Kim et al. (2019) examined 89 neurological patients with intervertebral disc disease (IVDD) who were referred to the Neurology Clinic at Konbuk University, South Korea, over 3 years. Kim et al.’s study averaged approximately 30 patients with IVDD per year, whereas the present study identified an average of only 4 patients with intervertebral disc herniation (IVDH) annually over an 8.5‐year period, indicating a lower incidence in this study. Importantly, Kim et al.’s study was conducted in a specialized neurology clinic, whereas the current study included a wider range of cases referred to a general veterinary clinic, which accounts for the lower number of IVDD patients observed here. Furthermore, while Kim et al. focused on one type of neurological disease, the present study investigated a broader spectrum of neurological conditions in dogs. Elbert et al. (2022) conducted a retrospective study involving 207 neurological patients with infectious agents over an 11‐year period (approximately 19 patients per year). In comparison, the present study identified and investigated a total of 136 neurological patients with infectious agents over 8.5 years (16 cases per year), which aligns relatively well with Elbert et al.’s findings. The population studied at the Small Animal Veterinary Hospital in Switzerland involved a retrospective analysis of 4497 dogs over an 11‐year period, identifying approximately 409 neurological patients annually—approximately eight times more than those examined in the present study (Fluehmann et al. 2006). These differences likely reflect variation in referral volume, regional dog population size, and availability of specialized neurology services, rather than fundamental differences in disease biology.

Our study revealed a significant age‐related increase in the prevalence of neurological diseases in dogs, ranging from 2.5% in dogs under 6 months of age to 8.8% in dogs over 60 months of age (5 years). These findings are generally consistent with those of Fluehmann et al. (2006), who reported the highest prevalence in dogs aged 7–9 years and the lowest in dogs under 1 year (Fluehmann et al. 2006). However, our results contrast with those of Gomez Flores et al. (2021), who reported the highest prevalence in dogs up to 3 years of age (Gómez‐Flores et al. 2021). Discrepancies between studies may be attributed to varying age classifications and study populations, differences in population structure, and regional variations in exposure to infectious and degenerative conditions. Although the absolute number of affected dogs was higher in younger age groups, the calculation of prevalence relative to the total number of referrals in each age category revealed a clear increase in relative prevalence with advancing age, supporting the role of cumulative exposure to risk factors and age‐related degenerative processes.

No significant sex‐based differences in the prevalence of neurological diseases were observed, with rates of 4.8% in males and 4.6% in females. This aligns with the findings from previous studies (Elbert et al. 2022; Fluehmann et al. 2006; Mayousse et al. 2017). These results suggest that sex alone is unlikely to represent a major independent risk factor for neurological diseases in dogs, although sex‐related behavioural or environmental exposures may still influence specific aetiologies such as trauma or infectious diseases.

Seasonal variations in the prevalence of neurological diseases were not evident in our study, with rates of 5% in spring, 4.1% in summer, 4.9% in autumn, and 4.4% in winter. A review of the literature revealed a paucity of studies specifically investigating the impact of seasonality on the prevalence of neurological diseases in dogs. The absence of a clear seasonal pattern suggests that most neurological disorders in this population are influenced primarily by host, environmental, and infectious factors that remain relatively constant throughout the year.

The prevalence of neurological diseases varied significantly among the dog breeds in this study. To ensure reliable data, analysis was restricted to breeds with more than 50 referrals. Kochi dogs presented the highest prevalence of neurological diseases (11.5%), followed by Pekingese (7.1%), Great Danes (5.4%), and German Shepherds (5.2%). These findings diverge from those reported by Fluehmann et al. (2006) and Elbert et al. (2022), who reported the highest prevalence of neurological diseases in mixed‐breed dogs (Elbert et al. 2022; Fluehmann et al. 2006). Gomez Flores et al. (2021) reported the highest incidence in unspecified breeds (25.53%), followed by French Poodles (20.2%) (Gómez‐Flores et al. 2021). Additionally, Kim et al. (2019) reported a high incidence of neurological diseases in Maltese dogs (24.4%) in South Korea. These differences may reflect regional variations in breed popularity, genetic susceptibility, and environmental exposures, as well as referral patterns to veterinary hospitals.

In our study, we identified the most common locations for neurological lesions as follows: prosencephalon, multifocal lesions, and the thoracolumbar spinal cord. These findings are consistent with those reported by Gomez Flores et al. (2021) and Fluehmann et al. (2006), who also identified the prosencephalon and thoracolumbar regions as common sites of neurological involvement (Fluehmann et al. 2006; Gómez‐Flores et al. 2021). Additionally, our findings align with those of Tipold (1995), who reported multifocal lesions as the most prevalent site in a retrospective study conducted in California (Tipold 1995). The prominence of prosencephalic involvement is likely related to the high frequency of seizure disorders and infectious encephalitis, particularly distemper, which commonly affects the forebrain.

The most common cause of neurological diseases in our study was infectious disease (32.2%), followed by unknown causes (15.9%), trauma (13.3%), and intervertebral disc disease (7.6%). These results partially align with those of Gomez Flores et al. (2021), who reported degenerative causes as the most common (44.2%), followed by infectious (25.3%) and traumatic (13.7%) causes. Our findings also exhibit some concordance with those of Elbert et al. (2022), who identified idiopathic and infectious causes as the most prevalent at the Athens Veterinary Diagnostic Laboratory (Elbert et al. 2022). However, our results differ from those of Fluehmann et al. (2006), who reported higher proportions of degenerative (38%) and lower proportions of infectious (14%) and idiopathic (13%) causes (Fluehmann et al. 2006). Among the infectious diseases, the most common cause was the neurological form of distemper, affecting 112 dogs (26.5%). The high prevalence of distemper in our study aligns with the findings from Tipold (1995), who reported that inflammatory encephalitis due to canine distemper was the most prevalent condition in a necropsy study (Tipold 1995). The elevated prevalence of distemper in our study may be attributed to its high incidence in the region (Mousafarkhani et al. 2023; Sarchahi et al. 2022). In contrast, a study by Mayousse et al. (2017) conducted in France reported that type 1 IVDH was the most common neurological disease among French bulldogs. Given that IVDH ranked fourth in prevalence in our study, our findings do not align with those of Mayousse et al. The high prevalence of neurological distemper observed in this study likely reflects regional differences in vaccination coverage, access to preventive veterinary care, and the presence of free‐roaming or working dog populations, reinforcing the importance of vaccination programs in reducing neurological disease burden.

In the present study, IVDH was diagnosed in 7.6% (32/422) of the dogs, with the thoracolumbar region being the most common site of involvement (46.9%, 15/32). The average age of onset for IVDH in this region was 3.4 years. These findings are partially supported by those of Gomez Flores et al. (2021), who reported an IVDH incidence of 52.3% in Guatemalan dogs, with the thoracolumbar region also being the most frequent site (34.1%) (Gómez‐Flores et al. 2021). However, our study revealed a lower overall incidence of IVDH. Conversely, Mayousse et al. (2017) reported a significantly higher IVDH incidence in French bulldogs (70.3%) (Mayousse et al. 2017). While this contrasts with our findings, the average age of onset for thoracolumbar IVDH in their study (4 years) was comparable to our results. The comparatively lower proportion of intervertebral disc disease observed in this population may be attributed to differences in breed distribution and the limited availability of advanced imaging such as MRI, which may lead to underdiagnosis of mild or ambiguous cases.

In the present study, 87.3% of the dogs diagnosed with neurological diseases were evaluated via only basic diagnostic modalities, including general physical examinations, neurological assessments, CBCs, radiography, and myelography. This observation aligns with the findings of Tipold (1995), who also reported that many neurological conditions were diagnosed without the need for specialized diagnostic equipment (Tipold 1995). These findings highlight the critical value of structured neurological examination and lesion localization in clinical decision‐making, particularly in resource‐limited settings. Practical and cost‐effective diagnostic strategies may include rapid immunochromatographic tests for distemper, complete blood count, and radiography as first‐line tools. Additionally, teleconsultation with referral neurology centres may provide valuable diagnostic support in complex cases when MRI or advanced imaging is unavailable. Nevertheless, limited access to MRI, CT, and PCR testing likely contributed to the underdiagnosis or misclassification of certain conditions, particularly neoplastic, inflammatory, and degenerative disorders.

The primary treatment modality employed in this study was corticosteroid therapy. These drugs were administered to nearly half of all treated patients, with a substantial proportion receiving corticosteroids as monotherapy. Among patients treated exclusively with corticosteroids and with available follow‐up data, a 64.7% improvement rate was observed. These findings suggest that corticosteroids are particularly effective in inflammatory, immune‐mediated, or compressive neurological conditions. However, treatment response likely varied depending on underlying aetiology and lesion location, and the absence of standardized treatment protocols may have contributed to variability in outcomes. These findings align with those of Tipold (1995), who reported corticosteroids as the most common treatment for steroid‐responsive meningitis‐arteritis, with a 53.1% success rate (Tipold 1995). Similarly, Kim et al. (2019) noted that anti‐inflammatory drugs were the most frequently used postsurgical treatment in their study conducted in Jeonbuk, South Korea. In addition to anti‐inflammatory medications, anticonvulsants were also commonly used and demonstrated favourable recovery rates in seizure‐related disorders (69%), supporting their continued role in managing epileptic conditions.

Unfortunately, prognostic data were unavailable for 116 patients (27.5%) because of incomplete medical records. Among the 306 patients with documented prognoses, outcome assessment revealed substantial variability, reflecting differences in disease severity, aetiology, and treatment response. Follow‐up assessments were conducted for 158 patients; however, follow‐up was not feasible for many patients due to logistical and compliance‐related challenges. Among patients with follow‐up evaluations, approximately 45% showed complete or partial recovery, which is comparable to previously reported recovery rates (Kim et al. 2019). However, because follow‐up data were available for only 37.4% of the cases, outcome estimates should be interpreted cautiously, as missing follow‐up data may introduce bias if cases with poorer outcomes were less likely to return for reassessment.

This study has several limitations. One limitation of our study is the absence of formal statistical corrections for multiple comparisons, such as the Bonferroni adjustment. Because this study evaluated the complete population of neurological cases referred during the study period, the primary analytical objective was descriptive epidemiology rather than hypothesis testing. Inferential analyses were conducted to explore potential associations, but these should be interpreted as exploratory and hypothesis‐generating rather than confirmatory. Consequently, emphasis should be placed on observed prevalence patterns and clinical trends rather than isolated statistically significant associations. Future prospective studies with standardized diagnostic protocols and systematic follow‐up procedures are warranted to validate and expand upon these findings.

A notable limitation of this study is the high proportion of cases lacking complete documentation, particularly for long‐term follow‐up and vaccination history. Vaccination status was missing in 33.9% of the cases, and follow‐up data were unavailable in 62.6% of the cases. To minimize bias, percentages were calculated using available‐case denominators for each analysis. Comparison of available demographic and clinical variables between cases with and without follow‐up did not reveal statistically significant differences. However, missing data may still influence reported prevalence and outcome estimates, particularly if missingness was associated with unmeasured factors such as disease severity, socioeconomic conditions, or owner compliance. Therefore, reported recovery and prognostic outcomes likely represent estimates based on the subset of patients with available data rather than the entire study population.

Neurological disorders in this study were primarily diagnosed using clinical examination and accessible diagnostic tools. While these methods are widely accepted and clinically useful, limited access to MRI, CT, and PCR testing likely reduced diagnostic precision in certain cases. Incorporating advanced imaging and laboratory diagnostics in future studies would improve etiological classification and diagnostic accuracy. However, the findings of this study demonstrate that reliable clinical diagnosis and management of many neurological diseases remain feasible using structured neurological examination and cost‐effective diagnostic approaches.

A potential limitation of our study is the possibility of bias in breed‐specific prevalence estimates due to the hospital‐based nature of our data collection. Although we calculated breed‐specific prevalence rates using the total number of referrals for each breed during the study period and excluded breeds with fewer than 50 referrals to minimize the impact of small sample sizes, certain biases may persist. These include referral bias, owner preferences, and regional differences in breed popularity, all of which can influence the observed distribution of neurological diseases among breeds. Furthermore, our study did not adjust breed representation against the broader dog population in the region, as such demographic data were unavailable. Importantly, while breed‐related prevalence was reported, we did not account for genetic predispositions or perform breed‐specific genetic analyses. Genetic factors may significantly influence susceptibility to certain neurological diseases, and the absence of such data limits the ability to distinguish environmental or referral biases from true genetic predisposition. Therefore, although our findings provide valuable insight into breed‐associated neurological disease patterns within our referral population, they should be interpreted with caution and may not be generalizable to the wider dog population without considering underlying genetic influences.

In this study, cases classified as having an ‘unknown cause’ were those for which the available diagnostic workup was incomplete and a definitive etiological diagnosis could not be established. This was often due to financial constraints, owner refusal, or limited access to advanced diagnostic modalities such as MRI, CT, or CSF analysis. Additionally, some cases exhibited atypical or non‐specific neurological signs that did not match established diagnostic patterns, or presented with multiple possible differential diagnoses without a clear conclusion. All such cases were managed symptomatically. We acknowledge that the lack of advanced diagnostics in these cases may have led to underdiagnosis or misclassification, representing a potential source of bias in our results. Additionally, our approach to confirming canine distemper relied on either PCR or rapid diagnostic kits. Due to affordability and accessibility limitations, not all clinically suspected cases underwent PCR, which may have led to underdiagnosis or misclassification, particularly in early or atypical cases. While all cases included in the ‘confirmed distemper’ group had at least one positive test result (PCR or rapid test), we acknowledge that variability in test sensitivity across sample types (e.g., conjunctival swab, blood, CSF) and lack of dual‐confirmation in many cases pose a potential source of diagnostic bias.

A limitation of this study is that, due to its retrospective design, treatment regimens were not fully standardized across all cases. Although core treatment principles were applied consistently, variability in drug selection, dosing, and supportive care may have influenced clinical outcomes. Prospective studies using standardized treatment protocols would allow more precise evaluation of therapeutic efficacy.

Finally, the study was conducted at a single veterinary teaching hospital in northeastern Iran, which may introduce referral bias. Therefore, the findings may not be fully generalizable to the broader dog population. Multicentre studies incorporating diverse populations would provide more representative epidemiological data.

## 5 Conclusion

Infectious diseases, especially canine distemper, trauma, and unknown causes, are the primary contributors to neurological disorders in this region. This study provides valuable insights into the current diagnostic and therapeutic approaches for canine neurological diseases, revealing that a significant majority of cases are diagnosed without specialized equipment. These findings suggest that corticosteroids are the most commonly employed treatment modality and demonstrate notable effectiveness, particularly for treating inflammatory neuropathies. However, challenges related to data collection and follow‐up assessments may impact the reliability of our results. Improved systematic follow‐up procedures could enhance our understanding of treatment outcomes and overall prognoses for canine neurological conditions.

## Author Contributions

Ali Asghar Sarchahi: conceptualization. Ali Asghar Sarchahi, Ali Behrouzian, Hadi Jabbari Noghabi: methodology. Ali Asghar Sarchahi: writing – original draft preparation. Ali Asghar Sarchahi, Ali Behrouzian, Hadi Jabbari Noghabi: writing – review and editing. Ali Asghar Sarchahi: supervision.

## Funding

This work was supported by the Research Council of Ferdowsi University of Mashhad under grant No. 61120.

## Ethics Statement

This research was approved by the Research Council of Ferdowsi University of Mashhad under grant No. 61120.

## Conflicts of Interest

The authors have no conflicts of interest to declare.

## Data Availability

The datasets used and analyzed in this study are available from the corresponding author upon reasonable request.

## References

[vms370952-bib-0001] Abdel‐Saeed, H. , and N. E. Elmashad . 2020. “Clinical and Diagnostic Studies on Common Causes of Canine Meningeo‐Encephalitis in Egypt.” International Journal of Veterinary Science 9: 429–432.

[vms370952-bib-0002] Bear, M. , B. Connors , and M. A. Paradiso . 2020. Neuroscience: Exploring the Brain, Enhanced Edition: Exploring the Brain. Jones & Bartlett Learning.

[vms370952-bib-0003] Elbert, J. A. , W. Yau , and D. R. Rissi . 2022. “Neuroinflammatory Diseases of the central Nervous System of Dogs: A Retrospective Study of 207 Cases (2008–2019).” The Canadian Veterinary Journal 63: 178–186. 35110776 PMC8759338

[vms370952-bib-0004] Ettinger, S. J. , E. C. Feldman , and E. Cote . 2024. Textbook of Veterinary Internal Medicine‐Inkling E‐Book: Ettinger's Textbook of Veterinary Internal Medicine‐eBook. Elsevier health sciences.

[vms370952-bib-0005] Fluehmann, G. , M. Doherr , and A. Jaggy . 2006. “Canine Neurological Diseases in a Referral Hospital Population Between 1989 and 2000 in Switzerland.” Journal of Small Animal Practice 47: 582–587. 17004950 10.1111/j.1748-5827.2006.00106.x

[vms370952-bib-0006] Gómez‐Flores, A. I. , J. J. Chávez‐López , and D. M. Villatoro‐Chacón . 2021. “Characterization of Neurological Diseases in Canines: University of San Carlos of Guatemala, Year 2017.” MVZ Cordoba 26: e2047.

[vms370952-bib-0007] Gülersoy, E. , C. Balıkçı , İ. Günal , et al. 2023. “Initial Clinical Manifestations of Dogs With Neurological Distemper.” Harran University Journal of the Faculty of Veterinary Medicine 12: 152–159.

[vms370952-bib-0008] Kim, D. , J.‐S. Kang , Y.‐U. Kim , D.‐B. Lee , S.‐Y. Heo , and N.‐S. Kim . 2019. “A Retrospective Study of Intervertebral Disk Disease Confirmed by MRI in Dogs: 89 Cases (2012‐2015).” Journal of Veterinary Clinics 36: 139–144.

[vms370952-bib-0009] Mayousse, V. , L. Desquilbet , A. Jeandel , and S. Blot . 2017. “Prevalence of Neurological Disorders in French Bulldog: A Retrospective Study of 343 Cases (2002–2016).” BMC Veterinary Research 13: 1–10. 28676057 10.1186/s12917-017-1132-2 PMC5497356

[vms370952-bib-0010] Mousafarkhani, F. , A. A. Sarchahi , H. Mohebalian , J. Khoshnegah , and M. Arbabi . 2023. “Prevalence of Canine Distemper in Dogs Referred to Veterinary Hospital of Ferdowsi University of Mashhad, Mashhad, Iran.” Veterinary Research Forum 14: 153–160. 37033774 10.30466/vrf.2022.541661.3269 PMC10073810

[vms370952-bib-0011] Sarchahi, A. A. , M. Arbabi , and H. Mohebalian . 2022. “Detection of Canine Distemper Virus in Cerebrospinal Fluid, Whole Blood and Mucosal Specimens of Dogs With Distemper Using RT‐PCR and Immunochromatographic Assays.” Veterinary Medicine and Science 8: 1390–1399. 35363942 10.1002/vms3.790 PMC9297742

[vms370952-bib-0012] Tipold, A. 1995. “Diagnosis of Inflammatory and Infectious Diseases of the central Nervous System in Dogs: A Retrospective Study.” Journal of Veterinary Internal Medicine 9: 304–314. 8531175 10.1111/j.1939-1676.1995.tb01089.x

